# Gut microbiota: A new target for T2DM prevention and treatment

**DOI:** 10.3389/fendo.2022.958218

**Published:** 2022-08-11

**Authors:** Lulu Liu, Jiheng Zhang, Yi Cheng, Meng Zhu, Zhifeng Xiao, Guangcong Ruan, Yanling Wei

**Affiliations:** ^1^ Department of Gastroenterology, Chongqing Key Laboratory of Digestive Malignancies, Daping Hospital, Army Medical University (Third Military Medical University), Chongqing, China; ^2^ Department of Plastic and Cosmetic Surgery, Daping Hospital, Army Medical University (Third Military Medical University), Chongqing, China

**Keywords:** gut microbiota, type 2 diabetes mellitus, bile acids, short-chain fatty acids, amino acids, fecal microbiota transplantation, probiotics, herbal medicines

## Abstract

Type 2 diabetes mellitus (T2DM), one of the fastest growing metabolic diseases, has been characterized by metabolic disorders including hyperglycemia, hyperlipidemia and insulin resistance (IR). In recent years, T2DM has become the fastest growing metabolic disease in the world. Studies have indicated that patients with T2DM are often associated with intestinal flora disorders and dysfunction involving multiple organs. Metabolites of the intestinal flora, such as bile acids (BAs), short-chain fatty acids (SCFAs) and amino acids (AAs)may influence to some extent the decreased insulin sensitivity associated with T2DM dysfunction and regulate metabolic as well as immune homeostasis. In this paper, we review the changes in the gut flora in T2DM and the mechanisms by which the gut microbiota modulates metabolites affecting T2DM, which may provide a basis for the early identification of T2DM-susceptible individuals and guide targeted interventions. Finally, we also highlight gut microecological therapeutic strategies focused on shaping the gut flora to inform the improvement of T2DM progression.

## 1 Introduction

T2DM is the most common metabolic disease and is mainly characterized by metabolic disorders, such as hyperglycemia, hyperlipidemia and IR. Diabetes can lead to various serious complications, such as coronary artery disease, lower extremity arteriopathy, retinopathy and diabetic nephropathy, which affect the life quality of a large populations worldwide (1, 2). The rapid growth of diabetes poses an alarming burden on global social and economic development (3). Recent data by the International Diabetes Federation (IDF) in 2021 have demonstrated that the global prevalence of diabetes in 20- to 79-year-olds was estimated at 10.5% (approximately 537 million people) and predicted to increase to 12.2% (783.2 million people) in 2045. The diabetes prevalence is projected to be higher in middle-income countries (21.1%) than in low-income countries (11.9%) and high-income countries (12.2%) between 2021 and 2045 (4, 5). The prevalence of T2DM is greater than 25% in older patients over 65 years of age; treatment of older patients is more difficult because complications are more likely to occur (6, 7). Today, the main treatment strategies for T2DM include surgery, pharmacotherapy, exercise therapy, diet, nutrition and multifactorial therapy (8, 9). The core of the treatment of T2DM is to solve the problem of hyperglycemia. The use of medication based on lifestyle modification is the cornerstone of the treatment of T2DM. If blood glucose remains uncontrolled, additional treatment with insulin and other injectable drugs will eventually be needed (10). Insulin injection therapy is the most effective way to control blood glucose (11). However, with increased IR, the risk of hypoglycemia is increased if insulin injections are increased, thus escalating the risk of cardiovascular-related complications. Therefore, the underlying mechanisms of T2DM need to be identified for early diagnosis and treatment.

Recognized risk factors for diabetes include obesity, poor dietary habits, positive family history and genetics (12, 13). A variety of factors play a critical part in the development of diabetes. Recently, plenty of studies have proposed a link of the intestinal microbiome and its metabolites with the metabolic health of the human body (14, 15). Genome-wide association reports show significant associations between specific gut microbes, bacterial genes and metabolic pathway variants in T2DM (16). The possible mechanisms of T2DM induced by the gut microbiota may be linked to IR, BA metabolism, disturbances of lipid metabolism and endotoxemia (17). Furthermore, many researches have shown that the relationship between BAs and microbiome plays a key role in the pathogenesis of metabolic disorders including T2DM (18, 19). The study showed that the imbalance in the interplay between BAs and the intestinal flora led to composition changes of the BA pool, as well as alterations in the intestinal flora structure and endocrine signaling pathways, which they suggest will influence the development of T2DM (20, 21). In addition, increased production of trimethylamine N-oxide (TMAO) and branched-chain amino acids (BCAAs) as well as changes in BA and SCFA metabolism may lead to changes in fat levels, thereby affecting insulin signaling (22–24).

Disorders of energy balance due to gut microbial dysbiosis have an integral role in the progression of T2DM (25). However, the mechanisms of intestinal flora in T2DM are not fully understood. Thus, the purpose of this review is to discuss the altered intestinal microbiota in T2DM and the role of metabolites in T2DM relative to a healthy human host. We provide a summary of microbiota-based therapeutic approaches to improve T2DM.

## 2 Association between intestinal flora and T2DM

The impact of societal advances and changes in people’s lifestyles, which have led to a greater intake of high-energy foods and a lack of physical activity, has left individuals in a suboptimal state of metabolic health with a high incidence of metabolic diseases, such as obesity, T2DM, nonalcoholic liver disease and cardiometabolic disease (CMD) (26, 27). The two main pathogenic mechanisms of diabetes are the relative lack of insulin secretion and IR, with IR being the main cause of T2DM. Diabetes biomarkers, such as venous glucose levels and glycated hemoglobin (HbA1c), have limitations as predictive biomarkers, especially in elderly or hyperlipidemic patients. Therefore, the identification of other early predictors is needed.

The collection of all gut microbial genes in an individual (i.e., the microbiome) represents a gene pool that is an order of magnitude higher than the human genome (28). Therefore, the intestinal microbiome is considered an “ organ” with important roles in enhancing host immunity, facilitating the digestion of food, regulating intestinal endocrine function, regulating neural signaling, modifying drug action, modifying metabolism and eliminating toxins. This symbiotic relationship with the host ensures proper development of the human metabolic system. Gut microbial metabolites absorbed by the host act on receptors in organs such as the liver, gut, brown adipose tissue (BAT), white adipose tissue (WAT) and the central nervous system (CNS), and they are involved in micronutrient synthesis, intestinal motility, mineral absorption and electrolyte absorption (29, 30). A growing number of studies have demonstrated a strong link between the intestinal flora and human disease, particularly its role in obesity and T2DM (17, 31–35). Improvement of insulin sensitivity by altering the composition of the intestinal microbiota has gained attention from many researchers, and gut flora therapy has become a new treatment modality.

## 3 Changes of intestinal flora in patients with T2DM and obesity

Approximately 86% of patients with T2DM are overweight or obese, and obesity is considered the greatest risk factor for T2DM (36), which is commonly used as an early warning marker for T2DM. Compared to healthy adults, obese and T2DM populations show significant changes in gut microbes and their metabolites (**Table 1**). Studies have shown that modern lifestyle changes and the use of antimicrobial drugs, among other factors, have led to a decrease in gut microbial diversity in many developed populations (72).

**Table 1:** Changes in intestinal flora and metabolites in T2DM and obesity.

Status	Gut bacteria(↑)	Gut bacteria (↓)	Changes in metabolites	Functional changes	Reference
**T2DM**	**——**	*Roseburia* *Subdoligranulum*	Degradation of Catalase and ribose, glycine and tryptophan amino acid ↑	Peroxide stress ↑ Inflammation↑ Insulin resistance	(37–40)
	**——**	*Akkermansia*	Butyrate and propionate production↓	Insulin resistance Insulin sensitivity↓ Appetite and body weight↑	(41, 42)
	*Proteobacteria* *Bacteroidetes* *R. gnavus*	*Anareotruncus colihominis, Butyrivibrio crossotus, Faecalibacterium*	Butyrate ↓ Mucus degradation ↑	Insulin resistance Inflammation↑	(39, 43–45)
	*Escherichia/* *Shigella*		BCAA↑	Insulin resistance	(46)
	*Prevotella copri*	**——**	SCFAs↓	**——**	(47)
	**——**	*Roseburia* *Ruminococcus* *Eubacterium*	SCFAs↓	**——**	(48)
	**——**	*Ruminococcus* *Bifidobacterium, Bacteroides, Clostridium, Eubacterium, Listeria*,	Secondary bile acids↓	Insulin ↓ Glucose sensitivity↓	(49, 50)
	*Prevotella copri* *Bacteroides vulgatus* *Streptococcus*	**——**	BCAAs↑	Insulin resistance	(17, 51, 52)
	**——**	*Lactobacillus* *Akkermansia*	SCFAs↓	Inflammation↑ Glucose and energy disorders	(39, 40)
	*Dorea*	*Akkermansia* *Parabacteroides* *Streptococcus* *Bifidobacterium*	Tyrosine and butyrate production↓	Insulin resistance Damage to the intestinal mucosal barrier	(53)
	**——**	*Bacteroidetes*	Sulphate reduction↑ Butyrate↓	Oxidative stress↑	(38)
	*Prevotella copri* *Bacteroides vulgatus*	*Butyrivibrio crossotus* *Eubacterium siraeum*	Increased plasma BCAA concentrations	Insulin resistance ↑ Aggravated glucose intolerance	(17, 54)
	*Streptococcus mutans* *Eggerthella lenta*	**——**	Imidazole ↑	Impair insulin signalling	(55)
	*Desulfovibrio* spp.	**——**	Promote interleukin6 (IL-6) and IL-8 secretion	Inflammatory response↑	(56)
	**——**	*A. muciniphila*		Glycolipid levels↑	(57)
**Obesity**		*Akkermansia*	SCFA↓	Insulin sensitivity ↓ Inflammation↑	(58, 59)
	*Firmicutes*	*Bacteroidetes*	**——**	Insulin resistance	(38, 60)
	*Bacteroidetes* *Firmicutes* *Betaproteobacteria*	**——**	BCAA↑	Insulin-resistant	(61, 62)
	*Bacteroides* *Parabacteroides*	*Turicibacteraceae* *Moryella* *Lachnospiraceae* *Akkermansia*	SCFA↓	Glucose and energy disorders	(63)
	**——**	*Ruminococcaceae* *Lachnospiraceae*	SCFAs	Fat accumulation	(64)
	**——**	*Ruminococcaceae* *Clostridia* *Christensenellaceae* *Dehalobacteriaceae* *SHA-98* *MethanobacteriaceaeRF39* *Oscillospira*	Acetate and butyrate↓	Affect energy metabolism Insulin sensitivity	(34, 65–68)
	**——**	*Eubacterium ventriosum* *Roseburia intestinalis*	SCFA↓	Insulin resistance Inflammation↑	(69)
	*Ruminococcus champaneliensis* *Prevotella copri*	*Bacteroides*	**——**	Fat accumulation	(70)
	**——**	*Firmicutes species* *Proteobacteria species*	LPS Amino acids Short-chain fatty acids↓	Insulin resistance	(71)

Meaning of symbols in the table. ↑, increase; ↓, decrease.

Studies have shown that individuals with low abundance of intestinal flora are predisposed to obesity, IR and dyslipidemia (43). A study of gut microbial diversity in patients with low gene count (LGC) and high gene count (HGC) has shown that *Lactobacillus*, *Prevotella*, *Bacteroides*, *Desulfovibrio* and *Oxalobacter* spp. in the intestinal microbiota are decreasing in abundance; this decrease in diversity may promote the development of metabolic disorders. Functional changes in the microbiota of individuals with LGC mainly include a reduction in butyrate-producing bacteria and an elevation in the ratio of *Akkermansia* to *R. torque/gnavus*, leading to enhanced mucus degradation, decreased hydrogen production potential, decreased methane production potential, increased *Campylobacter/Shigella* abundance and increased peroxidase activity (43). This metabolic disturbance caused by an imbalance between pro- and anti-inflammatory bacterial species in LGC individuals puts them at an increased risk for metabolic diseases, such as prediabetes and T2DM (73).

A previous study has shown that feces from obese twins transplanted into germ-free mice results in weight gain (61). Additionally, fecal transplantation studies have shown that obese subjects with metabolic syndrome have increased insulin sensitivity after the transfer of gut microbiota from a lean donor (37, 74, 75). These studies provide a theoretical basis for linking intestinal flora and whole-body energy metabolism, and they also highlight the important role of the intestinal flora in host metabolism (76, 77). Recent studies have suggested that the human intestinal microbiota is altered in obese individuals relative to lean individuals, probably primarily by an elevation in the obesity-associated *phylum Ligustrum* and a reduction in *Bacteroidetes (* 78, 79). A study of 416 twin pairs has shown that *Dehalobacteriaceae*, *Christensenellaceae* and SHA-98 were found to be significantly lower in the intestinal flora of obese individuals compared to those of lighter weight. The *Christensenellaceae* family is known to be producer of SCFAs (80). In addition, it has been demonstrated that *Christensenella minuta* amended and transplanted into recipient mice results in a tendency to lose weight. *Oscillospira* also produces SCFAs by degrading host blood glucose. In recent studies, we observed that *Oscillospira* and the methanogenic archaeon *Methanobrevibacter smithii* are abundant in healthy weight subjects and may promote leanness (65, 66, 81). Lean individuals also exist in T2DM (82). A study on the intestinal flora of lean diabetic patients has demonstrated that lean T2DM patients show a reduced *Akkermansia muciniphila* abundance, which is positively associated with a decrease in their insulin secretion (37). Studies have shown that *Ruminococcus*, *Clostridium*, *Bifidobacterium*, *Bacteroides*, *Eubacterium*, *Listeria* and others are the main intestinal microorganisms that affect the formation of secondary bile acids (49, 50). It has been shown that BAs modulate the metabolism and energy homeostasis of host by activating FXR receptors and TGR5, thereby shaping the intestinal flora (19).

Microorganisms including *Blastocystis* spp. and *Prevotella copri* have been found to be indicators of favorable postprandial glucose metabolism. And studies have shown that *Prevotella copri* supplementation improves glucose metabolism in mice (83). However, a study has demonstrated that *Prevotella copri* was associated with the production of BCAA, which is correlated with IR and glucose intolerance (17). It has been suggested that the elevated richness of *Bacteroides vulgatus* and *Prevotella copri* in IR individuals relative to the normal group may lead to an increased potential for BCAA synthesis; however, *Butyrivibrio crossotus* and *Eubacterium siraeum* abundance decreases, leading to reduced BCAA catabolism (17). Increased concentrations of BCAAs in circulation (including leucine, isoleucine and valine) may be biomarkers of IR and increased risk of T2DM (54).

Patients with T2DM are often treated with multiple drugs for glycemic control and prevention of cardiovascular complications. Thus, drug therapy usually affects the gut microbiota of patients, and the role of the gut microbiota may be confounded by the effects of various drugs (84). Therefore, many recent studies on T2DM flora have focused on the early stages of T2DM (not yet taking drugs). Although plasma glucose concentrations have not yet reached the diabetic range in the early stages of diabetes, individuals are at escalated risk of developing significant T2DM and cardiovascular disease (85). Alterations in the intestinal microbiota of unmedicated prediabetic patients have been reported to be dominated by butyrate-producing bacteria, *Akkermansia muciniphila*, and some bacteria with pro-inflammatory potential (86).

A previous study on the effect of metformin treatment on intestinal microbes in individuals with T2DM has reported a reduction in *Roseburia*, *Subdoligranulum* and a cluster of butyrate-producing *Clostridium* spp. in metformin-naïve T2DM patients, consistent with previous indications (37, 38). Metformin treatment modifies the intestinal microbiota in individuals with diabetes as indicated by enrichments in mucin-degrading *Akkermansia muciniphila* and SCFA-producing bacteria such as *Bifidobacterium bifidum* and *Butyrivibrio (* 87). An independent amplicon-based T2DM cohort analysis has similarly validated an elevation in *Escherichia coli* and a reduction in *Enterobacteriaceae* abundance in metformin-treated patients with several gut microbial genera more similar in abundance to normal control levels, particularly *Subdoligranulum* and *Akkermansia*. Therefore, we hypothesized that metformin may alleviate T2DM by affecting the microbiota and its microbial production of SCFAs. It has been shown that probiotic therapy based on flora modulation increases the richness of SCFAs while achieves a reduction in IR, and this therapy has been reported to have a similar effect under metformin treatment (41, 42, 88). Some studies have shown that probiotics act as adjuvants to metformin by increasing the production of butyrate, thus allowing enhanced glucose management (89).

All of these findings provide strong evidence that the gut microbiome can be used to improve the prediction of metabolic diseases, such as T2DM, and to discover new targets for diabetes treatment. In this review, we focus on how the intestinal microbiota and its metabolites affect the pathogenesis of T2DM. We summarize examples of microbiota-targeted interventions for T2DM and highlight the great promise of this therapeutic approach in the field of T2DM research.

## 4 Relationship between gut microbial metabolites and T2DM

### 4.1 BAs

**Figure 1:**
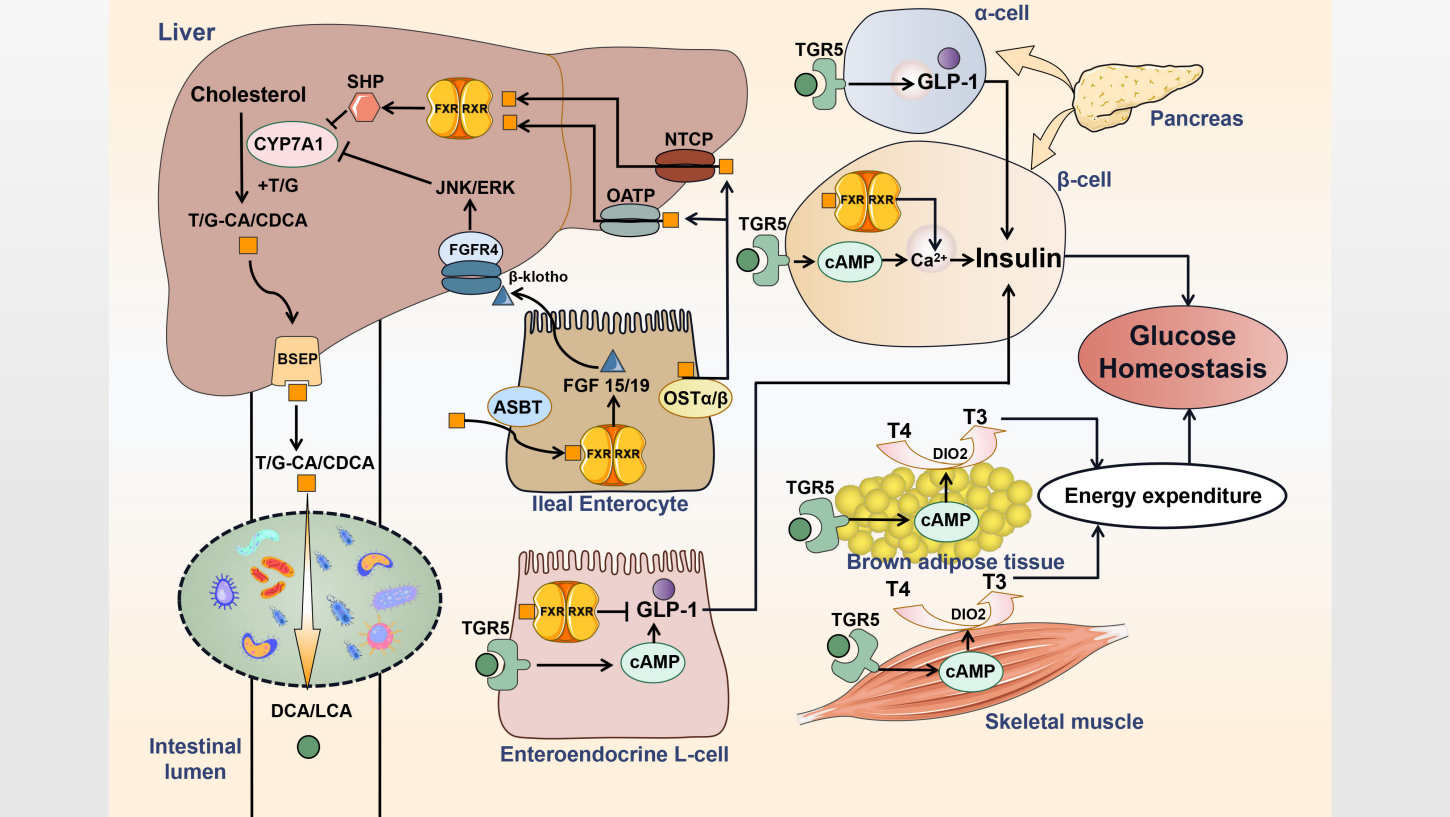
Interactions of BAs and gut microbe influence host metabolism. Primary bile acids are synthesized and then conjugated with taurine or glycine in hepatocytes. Conjugated bile acids are transported into the bile duct by BSEP. Most conjugated bile acids are reabsorbed *via* ASBT and circulate to the liver by OSTα/β, OATP and NTCP, while a small part is converted into secondary bile acids by deconjugation and dehydroxylation of gut flora. Bile acids acts as the endogenous ligands for FXR and TGR5 to generate distinct effects on metabolism regulation. T, taurine; G, glycine; CA, cholic acid; CDCA, chenodeoxycholic acid; DCA, deoxycholic acid; LCA, lithocholic acid; BSEP, bile salt export protein; FGF, fibroblast growth factor; FGFR, FGF receptor; RXR, retinoid X receptor; SHP, small heterodimer partner; JNK, c-Jun N-terminal kinase; ERK, extracellular signal-regulated kinase; OST, organic solute transporter; OATP, organic anion-transporting polypeptide; NTCP, sodium taurocholate cotransporting polypeptide; ASBT, apical sodium-dependent bile acid transporter; DIO2, type 2 iodothyronine deiodinase; T4, thyroxine; T3, thyroid hormone.

In the liver, BAs are synthesized from cholesterol and act as promoters for emulsification, absorption and transportation of dietary lipids as well as fat-soluble vitamins in the intestinal lumen. BAs are mainly metabolized by the actions of intestinal bacteria, which play a critical role in glucose regulation. BAs also act as regulators of intestinal microbiota as well as signaling molecules that modulate metabolic homeostasis (**Figure 1**). Interactions of BAs and intestinal flora have a profound impact on IR and the progression of T2DM.

#### 4.1.1 Synthesis of BAs

Cholesterol 7α-hydroxylase (CYP7A1) predominantly regulates the classic pathway (namely, the neutral pathway) of BA synthesis, while the alternative pathway (namely, the acidic pathway) depends mainly on CYP27A1. CYP8A1 and CYP7B1 also play distinct roles in the process (49). Glucose and insulin act as the main postprandial factors inducing CYP7A1 gene expression as well as BA synthesis (90). Mechanistically, glucose induces CYP7A1 gene transcription by increasing ATP to reduce the AMP/ATP ratio, which inhibits AMP-activated protein kinase (AMPK) activity, or by epigenetically modifying the acetylation state of the CYP7A1 chromatin structure (91). Cold exposure may also trigger hepatic conversion of cholesterol to BAs through the alternative pathway by induction of CYP7B1, leading to increased plasma levels and fecal excretion of BAs accompanied by alterations in intestinal microbiota that are characterized by higher abundance of *Deferribacteraceae* and *Lachnospiraceae* as well as lower abundance of *Porphyromonadaceae* and *Clostridiales*. Increased energy expenditure is also related to hepatic CYP7B1 induction, which is reduced in individuals with T2DM, suggesting that the alternative pathway plays a role in metabolic homeostasis (92). Another regulator of BA composition, CYP8B1 (an 12α-hydroxylase) has been reported to contribute to metabolic anomalies in T2DM as greater 12α-hydroxy/non12α-hydroxy BA ratios are correlated with decreased insulin sensitivity (93).

#### 4.1.2 BSHs and HSDHs in gut flora

Hepatocytes conjugate bile acids with taurine or glycine to increase their solubility before secretion (18). In the distal ileum, roughly 95% of conjugated bile acids (CBAs) are reuptaken *via* the apical sodium-dependent BA transporter (IBAT or ASBT) in conjugated form, while a small portion are deconjugated by the gut microbiota prior to resorption or converted into secondary bile acids (DCA from CA and LCA from CDCA, respectively), which are either absorbed through passive diffusion or excreted in the feces (94). BAs absorbed recirculate through the portal vein to the liver, where they are conveyed into hepatocytes to be reconjugated and then resecreted with newly synthesized BAs (95).

Microbial deconjugation refers to enzymatic hydrolysis by bile salt hydrolases (BSHs), namely, the removal of taurine or glycine conjugates, and it impedes active reabsorption of BAs *via* ASBT in the small intestine. In the human intestinal microbiome, BSHs are distributed among various microbial species and were identified early in several microbial genera, such as *Lactobacillus (* 96–99), *Clostridium (* 100), *Bifidobacterium (* 101–103), *Listeria (* 104, 105), *Enterococcus (* 106) and *Bacteroides (* 107). BSH activity exerts beneficial effects on bacteria by strengthening resistance to CBAs, assisting surviving in the gastrointestinal environment without causing bacterial overgrowth or damage to the host. This may facilitate colonization and development of the gut microbiota (108). High levels of BSH have also been reported to decrease body weight gain and plasma cholesterol in conventionally raised mice (109). However, some controversial results have suggested that deconjugated bile acids (DBAs) with higher binding affinity to farnesoid X receptor (FXR) may suppress the transcription of CYP7A1 and reduce BA synthesis from cholesterol (110). Moreover, some studies have deviated from previous concepts, indicating that the intrinsic chemical features and enzymatic preferences of various BSHs change the *Lactobacillus* transcriptome in a BA-dependent manner and contribute to the toxicity during *Lactobacillus* growth (111). Therefore, the importance of BSHs classification is highlighted as distinct BSHs in the identical *Bacteroides* strain differ in deconjugation ability. Generally, BSHs benefit metabolism, but the higher relative abundance (RA) of BSH phylotype with high activity may exert detrimental effects. To explore the relationship between the RA of BSHs and disease, it is important to first assess activity to identify particular BSHs rather than use an estimated RA of total BSHs (112).

BAs are C-24 steroids hydroxylated at the C-3, C-7 position, and also the C-12 position in the case of CA (113). 7α/β-dehydroxylating bacteria play an important role in transformations of BAs as the pool of secondary bile acids mainly consists of 7α/β-dehydroxylated BAs (e.g., LCA and DCA) (114). Different from deconjugation, BA 7α/β-dehydroxylation is confined to a limited number of gut bacteria, which lead to the salvage of BAs that evade active reuptake in the distal ileum, as deconjugation and 7α/β-dehydroxylation increase Pka and hydrophobicity of BAs to further permit passive absorption across the colonic epithelium (115). Because 7α/β-dehydroxylation is restricted to free bile acids, deconjugation is a prerequisite. 7α/β-Hydroxysteroid dehydrogenases (7α/β-HSDHs) have been identified in gut bacteria such as *Clostridium (* 116–118), *Bacteroides (* 119–121), *Escherichia (* 122–124) and *Eubacterium (* 125–127). While 7α-dehydroxylation functions as the most quantitatively vital bacterial biotransformation of BAs, 7β-dehydroxylation appears to be not essential in the human colon (128).

In diabetic patients, the BA pool composition and the associated pathways of biosynthesis are altered, which may involve a higher conversion of primary bile acids to secondary bile acids by the gut microbiota (129). Cirrhotics show lower abundance of *Ruminococcaceae*, *Lachonospiraceae* and *Blautia* (7α-dehydroxylating bacteria) while increased *Enterobacteriaceae* (potentially pathogenic), resulting in decreased conversion of primary to secondary fecal BAs (130). *Clostridium scindens* with 7α-HSDHs activity strengthens resistance to *Clostridium difficile* infection in a secondary bile acid-dependent manner upon administration (131). In addition to 7α/β-dehydroxylation, 3β-HSDH is identified in *Odoribacteraceae* strains to produce isoalloLCA, which exerts antibacterial effects against Gram-positive pathogens including *Enterococcus faecium* and *Clostridioides difficile* to maintain the intestinal homeostasis (132). 12α-dehydrogenation is involved in synthetic process from CA to ursodeoxycholic acid (UDCA), which functions in the therapy of certain gastrointestinal tract diseases, and contributes to counter the effects of LCA and DCA (133). *Clostridium scindens*, *Clostridium hylemonae* and *Peptacetobacter hiranonis* are identified as 12α-HSDH expressing strains (134). A new fecal isolate, Eggerthella lenta strain C592, is found to be a critical microbe in BA metabolism and express 3α-, 3β-, 7α-, and 12α-HSDHs (135, 136). 12β-HSDH interconverts the 12-oxo bile acids to the 12β-configuration to completes the epimerization, forming less toxic and more hydrophilic bile acids. 12β-HSDH activity is proved in *Clostridium paraputrificum (* 137).

The effects of acarbose are based on the gut microbiota-plasma BA axis as acarbose elevates the RA of *Bifidobacterium and Lactobacillus* while depleting *Bacteroides*, leading to altered RA of microbial genes that are responsible for BA metabolism. The results of acarbose treatment depend on microbiota compositions in the gut before treatment, and diverse abilities of the gut flora to metabolize BAs result in distinguishing therapeutic effects (138). Antibiotics modify the intestinal microbiota to change high-fat diet (HFD)-driven inflammatory signaling and BA composition, resulting in improved glucose metabolism and IR. These effects depend on interactions with the host’s systemic inflammatory response and genetic background (139).Patients undergoing bariatric surgery (especially gastric bypass procedures) show improvements in insulin sensitivity, which are correlated with increased BA concentrations in circulation and alterations in intestinal microbiome, indicating that elevations in circulating BAs and the interaction of BAs and gut flora following bariatric surgery as well as calorie restriction and body mass loss lead to T2DM remission (140).

#### 4.1.3 Targets of BAs

FXR, which is mainly activated by the primary BA CDCA, plays a critical role in modulating glucose homeostasis as well as insulin sensitivity (141, 142). In ileal enterocytes, activation of FXR promotes transcription of FGF19 (FGF15 in mouse), which circulates through the portal vein to the liver and further activates JNK/ERK signaling by binding to FGFR4/β-klotho heterodimer complex to inhibit expression of CYP7A1 in hepatocytes (143). In the liver, FXR activation not only induces small heterodimer partner (SHP) expression to inhibit CYP7A1, but also negatively interferes with glycolysis by inhibiting the carbohydrate response element binding protein (ChREBP), which is responsible for the expression of hepatic glycolytic genes (144–146). In intestinal L cells, FXR interacts with ChREBP to inhibit proglucagon mRNA levels induced by glucose. The effects of FXR on ChREBP also decrease intracellular ATP levels by inhibiting the transcription of glycolytic enzymes, resulting in a reduction in ATP-dependent Glucagon-like peptide-1 (GLP-1) secretion (147). In pancreatic β cells, FXR activation has been suggested to inhibit the K_ATP_ current and increase the cytosolic Ca^2+^ concentration, eventually resulting in increased insulin secretion (148, 149). These observations highlight the importance of the FXR-dependent control of BAs and glucose homeostasis.

In T2DM individuals, metformin decreases the abundance and BSH activity of *Bacteroides fragilis* to increase intestinal levels of glycoursodeoxycholic acid (GUDCA), which has been identified as a novel endogenous FXR antagonist in the gut. This finding demonstrates that metformin improves glucose metabolic dysfunction *via* a *Bacteroides fragilis*-GUDCA-intestinal FXR axis (150). In NAFLD patients with T2DM, obeticholic acid (OCA, an FXR agonist) activates FXR to mediate an increase in FGF19, resulting in weight loss and improved insulin sensitivity, accompanied by decreased endogenous BA production and 7a-hydroxy-4-cholesten-3-one (C4, a biomarker for BA synthesis) levels (151). Although serum levels of BA and C4 vary significantly among individuals, C4 plasma levels are considerably elevated in patients with T2DM and metabolic syndrome (MetS) (152). Studies in FXR-deficient mice fed a HFD have demonstrated that intestinal flora takes part in the development of IR and obesity by modulating BA and FXR signaling, and conversely, FXR may contribute to adiposity increase by changing the composition of gut microbiome, which is characterized by elevation in *Bacteroidetes* and reduction in *Firmicutes (* 153).

TGR5, a transmembrane G protein-coupled receptor, is activated mainly by the secondary bile acids, DCA and LCA (154). Previous studies have shown that TGR5 activation results in increased intracellular cyclic AMP (cAMP), leading to the maintenance of glucose homeostasis, preservation of pancreatic function and insulin sensitivity as a consequence of increased GLP-1 secretion and energy expenditure induced by enhanced mitochondrial function (155). In enteroendocrine L cells, activation triggers an increase in the synthesis and release of GLP-1. In systemic circulation, BAs escaping hepatic clearance activate TGR5 in BAT and muscle to activate type 2 iodothyronine deiodinase (DIO2), which converts inactive thyroxine (T4) into active thyroid hormone (T3) to promote energy expenditure (156). In addition, TGR5 positively regulates insulin secretion *via* a cAMP/Ca2^+^ pathway in pancreatic β cells, while it reprograms pancreatic α cells to produce GLP-1 to mediate the proliferation and mass of β cells (157, 158).

The FXR agonist, fexaramine (FEX), activates FXR to shape the gut flora inducing *Bacteroides* and *Acetatifactor*, which convert CDCA and UDCA to LCA. LCA not only activates TGR5 and further stimulates L cells to secrete GLP-1, resulting in improved insulin sensitivity, but also induces WAT browning by activating TGR5/cAMP signaling to improve energy metabolism and ultimately reduce weight (159). Wu et al. demonstrated that ablation of intestinal HIF-2a leads to an elevation in *Ruminococcus torques* abundance and a decrease in *Bacteroides vulgatus* by reducing lactate synthesis, resulting in elevated levels of taurine-conjugated cholic acid (TCA) and DCA. These changes activate adipose TGR5 to upregulate WAT thermogenesis, ultimately improving obesity and IR (160). Cholic acid-7-sulfate (CA7S), an endogenous BA sulfated metabolite, has been identified as a potent gut-restricted TGR5 agonist that induces the secretion of GLP1 from enteroendocrine L cells. CA7S is increased in the gastrointestinal tract after sleeve gastrectomy (SG), exhibiting anti-diabetic effects by enhancing glucose tolerance and reducing blood glucose levels (161). LCA has been identified to mediate the synthesis of CA7S in hepatocytes to influence host metabolism. Changes in the gut flora post-SG, particularly a decline in *Clostridia*, lead to a reduction in the production of LCA, resulting in increased production of CA7S (162). As CA7S is generated through microbiome-dependent signaling, its stability may differ among individual gut microbiomes (163).

GLP-1 is a proteolytic product of the proglucagon gene and regulates glucose homeostasis in the body. GLP-1 reduces appetite and slows gastric emptying in response to ingestion, and it acts on the pancreas to reduce glucagon secretion. GLP-1 also promotes glucose uptake and storage in muscle and adipose tissues while inhibiting glucose production in the liver (164, 165). Moreover, GLP-1 activates the GLP-1 receptor (GLP-1R) to promote proliferation and inhibit apoptosis of islet β cells to increase insulin biosynthesis and secretion (166). In T2DM patients, postprandial BA concentration is higher, and BA metabolism is upregulated, which may be due to the advantage of glucose-induced BA stimulation over other inhibiting factors to maintain GLP-1 levels as a compensation. Thus, the requirement of higher BA levels to stimulate enteroendocrine L cells to release GLP-1 as resistance to BA in L cells is suggested (167). Recently, studies have found that *Akkermansia muciniphila* secretes P9, a novel protein identified to induce the secretion of GLP-1 (168, 169).

### 4.2 SCFAs

The fermentation of undigested dietary components (e.g. fibre and resistant starch) in the large intestine mainly produces three SCFAs including acetate, propionate as well as butyrate, and various biochemical pathways are involved in the process (170). Thus far, as the most well studied metabolites of gut flora, SCFAs control immunomodulatory functions, promote the integrity of intestinal epithelium, as well as regulate insulin secretion and the proliferation of pancreatic β cell, playing a variety of roles in IR and T2DM (171). Most enteric bacteria are identified as acetate producers such as *Akkermansia muciniphila*, *Blautia hydrogenotrophica*, *Clostridium* spp., *Ruminococcus* spp., *Prevotella* spp., *Bifidobacterium* spp., *Bacteroides* spp., *Streptococcus* spp. Propionate producers include *Roseburia inulinivorans*, *Phascolarctobacterium succinatutens*, *Megasphaera elsdenii*, *Coprococcus catus*, *Ruminococcus obeum*, *Bacteroides* spp., *Dialister* spp., *Veillonella* spp., while *Roseburia* spp., *Anaerostipes* spp., *Faecalibacterium prausnitzii*, *Coprococcus catus*, *Coprococcus eutactus*, *Coprococcus comes*, *Eubacterium hallii*, *Eubacterium rectale* are proved to be burytate producers (170, 172, 173).

SCFAs exert benefits by activating G-protein coupled receptors (GPCRs) such as GPR41, GPR43 and GPR109A (174). Acetate, as well as propionate promotes GPR43 and GPR41 to release peptide YY (PYY) and GLP-1 to influence satiety and intestinal transit (175). Butyrate has also been proved to induce GLP-1 and PYY to increase insulin secretion and maintain glucose homeostasis (176, 177). In addition, butyrate not only activates GPR109A and inhibits histone deacetylases (HDACs) to exert anti-inflammatory effects, but also produces antimicrobial peptides to promote epithelial barrier function (178). Another study has shown that butyrate induces satiety to reduce food intake and activate BAT to promote fat oxidation by influencing the gut-brain neural circuit (179). A study suggested that butyrate produced in the gut mainly improves dynamic insulin response to food ingestion, instead of maintaining glucose homeostasis in the fasted state, and increased butyrate production driven by a genetically influenced change in the gut microbiome is beneficial for pancreatic β-cell function (180). Furthermore, butyrate was reported to maintain the gut microecological balance by limiting the bioavailability of respiratory electron acceptors of *Enterobacteriaceae* in the colonic lumen and thus preventing a dysbiotic proliferation of opportunistic pathogenic *Salmonella* and *Escherichia (* 181). *Dysosmobacter welbionis* J115^T^, a new butyrate-producing bacterium belonging to the *Ruminococcaceae* family and isolated from human feces, is identified to be negatively correlated with fasting plasma glucose, HbA1c and BMI in overweight or obese individuals with metabolic syndrome, and influences host metabolism in beneficial and direct way (182). However, another study highlighted the importance of functional dysbiosis of gut flora in association with T2DM pathophysiology, rather than a specific microbial species. They found only a mild degree dysbiosis of gut flora in T2DM patients, while a decrease in butyrate-producing bacteria, which seemed to be metabolically beneficial (38). In T2DM patients, reduced SCFA levels caused by decreased abundance of SCFA-producing bacteria may contribute to the progression of IR and T2DM (31). Yet animal and clinical researches have demonstrated that the levels of fecal SCFA are positively correlated with IR and body weight (183), controversies over the role of SCFAs in T2DM and IR still remain, and further investigations are required.

### 4.3 AAs

Research has shown that elevated concentrations of AAs, particularly BCAAs, are correlated with increased IR and risks of T2DM (184). BCAAs also promote the development of IR associated with obesity in a setting of a HFD (185). The intestinal microbiota acts as an independent factor of increased serum BCAA levels in individuals with IR. *Prevotella copri* and *Bacteroides vulgatus* have been identified as the major gut microbes correlated with the biosynthesis of BCAAs and IR (17). Among BCAAs, a previous study has further identified a critical role of isoleucine in metabolic health as a diet with low isoleucine activates the FGF21-UCP1 axis to reprogram the metabolism of adipose tissue and liver, improving energy expenditure and hepatic insulin sensitivity. Reducing dietary valine induces similar but milder metabolic effects, whereas low leucine does not show these effects, thus suggesting that decreasing dietary isoleucine is important to prevent and treat diabetes (186). Of note, IR may induce the protein degradation normally suppressed by insulin and impair the oxidative metabolism of BCAA to result in aminoacidemia, suggesting that increased BCAA may be a marker of insulin action loss instead of a causation (187).

Similar to BCAAs, aromatic amino acids (AAAs) also take part in the pathogenesis of hyperglycemia as the metabolisms of BCAAs and AAAs change prior to alteration of glucose homeostasis (188). BCAAs and AAAs both act as markers of IR development in young adults with normoglycemia, suggesting that their correlation with diabetes risk partly depends on IR (189). *Clostridium sporogenes* has been identified to metabolize all three AAAs in the human gut (190). Ka et al. demonstrated that excessive consumption of monosodium glutamate (MSG) positively correlates with overweight and BMI among healthy Chinese adults, suggesting a correlation between glutamate and obesity (191). In obese individuals, Liu et al. observed a decrease in *Bacteroides thetaiotaomicron* abundance, which is negatively correlated with serum levels of glutamate. These microbial and metabolic changes are partly reversed by bariatric surgery, and the reduced glutamate levels in circulation contribute to the improvement of IR and hyperglycemia (81).

### 4.4 Others

Gut flora convert choline in diet to trimethylamine (TMA), the precursor of TMAO. A high-fat diet elevates choline catabolism of *Escherichia coli* by changing the physiology of colonic epithelium, thus increases TMAO levels in circulation (192). Intestinal microbiota (predominantly *Enterobacteriaceae*) also reduces TMAO to TMA, which is untaken by the host and circulated to the liver to be further converted into TMAO by hepatic enzymes. Conversely, TMAO influences the metabolism and growth of intestinal microbiota in a taxon and source-dependent manner (193). Urocanate produced by histidine metabolism is converted to imidazole propionate by microbiota expressing urocanate reductase. T2DM patients show higher concentrations of imidazole propionate in peripheral and portal blood than healthy individuals, indicating that imidazole propionate may impair insulin signaling. *Eggerthella lenta* and *Streptococcus mutans* were identified to produce imidazole propionate, which was proved to aggravate glucose intolerance in mice (55). In addition, elevated serum levels of imidazole propionate are correlated with decreased abundance of intestinal flora (194). Gut flora-produced indolepropionic acid, a promising biomarker for T2DM progression, is suggested to be positively correlated with insulin secretion and negatively associated with low-grade inflammation as it preserves function of islet β cells (195).

## 5 Therapeutic options for targeting the gut microbiota in T2DM and obesity

**Figure 2:**
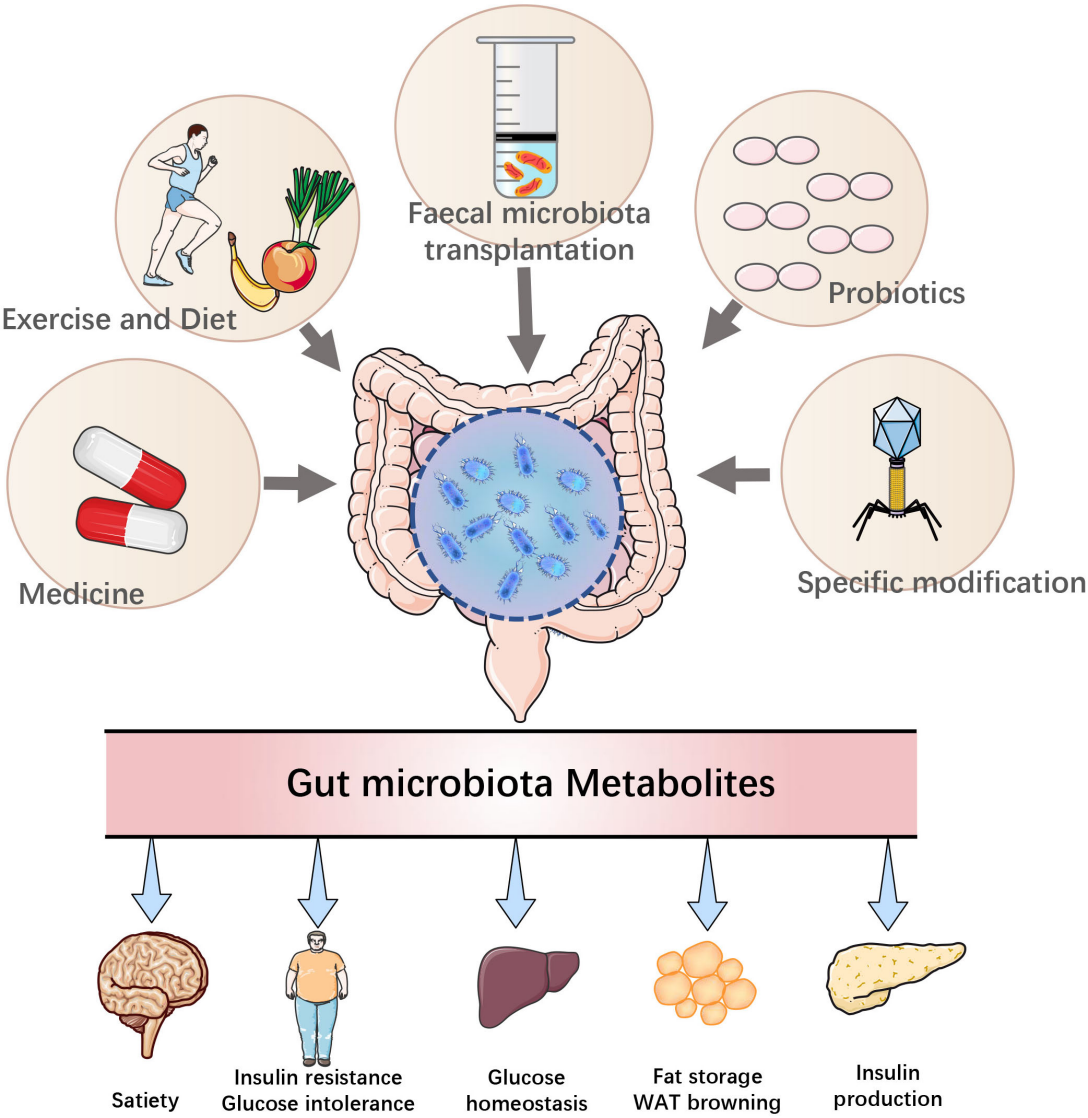
Modulation of T2DM by therapeutic approaches targeting the gut microbiota.

In summary, the intestinal microbiota and its metabolites are involved in the occurrence and progression of T2DM, and the process can be reversed to some extent by regulating the intestinal microbiota and its metabolites, providing a new idea for clinical treatment (196) (**Figure 2**).

### 5.1 Probiotics

When applied in appropriate amounts, probiotics, which are described as the living microbes, provide the host with health benefits (197). Studies have shown that the administration of probiotics regulates intestinal flora, suppresses inflammatory responses, improves intestinal barrier function, antagonizes each other with pathogens and produces beneficial microbial metabolites, including SCFAs and BAs (198). Studies on the molecular mechanisms of probiotic intervention in T2DM have shown that probiotics have ameliorative effects on IR and hyperglycemia (199).

Studies have shown that *Akkermansia muciniphila* treatment improves liver function, reduces oxidative stress and inhibits inflammation in diabetic mice. *Akkermansia muciniphila* supplementation reduces chronic low-grade inflammation and increases anti-inflammatory factors, such as α-tocopherol and β-sitosterol (200). Glucose tolerance and insulin sensitivity in diabetic mice were also ameliorated by supplementation with *Lactobacillus mucilaginosa*. In addition, it has been shown that supplementation with *Lactobacillus acidophilus* improves intestinal barrier function, and a reduced inflammatory response in the liver and colon has been observed in animal models of diabetes. Studies have shown that *Akkermansia muciniphila* strains are not even required to colonize the intestine to exert beneficial metabolic health effects (201, 202). In addition, 14 probiotics were isolated from fermented camel milk, and the effects of these 14 probiotics encompassed an increase in SCFA, an improvement in function of intestinal barrier, an upregulation of GLP-1 secretion, all of which showed significant improvements in blood glucose in db/db mice (203). A randomized, double-blinded, placebo-controlled study has reported that the administration of probiotic improves glucose metabolism in T2DM individuals, while the consumption of fermented milk results in other metabolic alterations including an elevation in acetic acid and a reduction in inflammatory cytokines (TNF-α and resistin) (204).The role of probiotics in T2DM deserves recognition, but the FDA and EFSA do not yet have any approval statements for probiotics (205–207). To verify the effectiveness of probiotics, more clinical trials are required.

### 5.2 Fecal microbiota transplantation (FMT)

Microbiologists have isolated many probiotic bacteria in the last century, and although studies have demonstrated their efficacy in standard animal models, single microorganisms are weak in preventing and treating human diseases (208, 209). Consequently, their clinical benefits are limited (210). Therefore, it is necessary for multiple microbes to work together to remodel the intestinal microbiota. In recent years, FMT has received increasing attention in the field of biomedical and clinical medicine (211). FMT refers to the transfer of fecal microbiota from a healthy donor to an unhealthy recipient *via* capsules with freeze-dried stool (or other ways), it may correct dysbiosis by increasing microbial diversity and restoring microbial function (212).

Official guidelines have ratified FMT as a standard treatment for recurrent *Clostridium difficile* infection (CDI) (213). According to data available on clinicaltrials.gov, ongoing trials on FMT are focused on indications other than CDI, and many clinical trials have shown that it also has strong potential in the treatment of refractory ulcerative colitis, Crohn’s disease, irritable bowel syndrome and other intestinal diseases as well as many extraintestinal diseases, including diabetes, cancer, cirrhosis, entero-brain disease and other metabolic diseases (214–217).

Early studies have shown that germ-free mice receiving samples from obese donors gained more weight than those from lean suppliers (78). After transplantation of Kazaks normal glucose tolerance (KNGT) fecal bacteria to db/db mice, the richness of *Clostridium coccoides* and *Desulfovibrio* in the intestine markedly declined, while the fecal abundance of *Akkermansia muciniphila* are increased. Moreover, the levels of fasting blood glucose and postprandial glucose are significantly downregulated in db/db mice, while the levels of HDL-cholesterol are upregulated, suggesting that fecal bacteria from KNGT may be a promising source for treating T2DM individuals with FMT (218, 219).

Previous studies have demonstrated that BA concentrations are changed in T2DM patients (147, 220). In addition, a study on the functional identification of bacterial and viral genes before and after FMT has shown that metabolic pathways such as the degradation of fluorobenzoate and the biosynthesis of secondary bile acids were significantly altered. A recent study has shown that the composition of the intestinal flora affects metabolism of BAs (211). The formation of BAs, SCFAs, and intestinal transit time of the organism are altered after FMT treatment. A previous study showed that allogenic FMT using feces from post-Roux-en-Y gastric bypass donor (rYgB-D) exerted short-term effects on metabolism of glucose, adipose tissue inflammation and intestinal transit time in obese and treatment-naive male individuals with IR, compared with feces from a metabolic syndrome donor (MetS-D) (74, 212).

Some studies have shown high interindividual variability in strain-level outcomes following FMT (221). Changes in plasma metabolites are also reported. The improvement in glucose metabolism, and the regulation of gut microbiota and plasma metabolites by FMT from lean donors are dependent on the reduction in fecal microbial diversity at baseline. Thus, pretreatment fecal microbiota characteristics may differ in response to bacterial species of lean donors, and thus pretreatment status may be correlated with the treatment effects (44, 58, 74).

However, some contradictory results have been reported. A 12-week double-blinded randomized placebo-controlled pilot trial of oral FMT capsules has shown that weekly treatment of FMT capsules to obese adults led to intestinal microbiota transplantation in most recipients for at least 12 weeks. Despite the successful implantation of the colonies, obviously clinical metabolic effects were not observed during the study period (222). Because researchers tend to publish studies with positive data, it makes it more difficult to determine the actual results. Studies have shown that performing FMT is also risky, and that harmful microorganisms may be transferred to the recipient *via* FMT. A previous study showed that an individual receiving feces from an overweight but healthy donor had developed new-onset obesity. Therefore, it is essential to publish studies with negative data so that we can have an unbiased understanding of the intestinal microbiota and its role in diseases (223).

To explore the potential of FMT in metabolic diseases (T2DM), further detailed work is needed, such as determining the indications for recipients, optimal donor microbiome profile and appropriate dose frequency. Modulation of the gut microbiota by techniques including FMT may become a potential therapeutic option for T2DM management.

### 5.3 Herbal medicines

There is growing evidence that many herbs or their herbal compounds may have a therapeutic effect on T2DM by modulating the intestinal microbiota. Radix scutellariae can eliminate heat and dampness, cure jaundice and quench thirst. A modern pharmacological study has shown that scutellaria achieves hypoglycemic and lipid-regulating effects by reducing intrahepatic cholestasis or increasing BA excretion in the feces (224). Studies have shown that baicalin improves diabetes by modulating the interplay between BAs and intestinal flora, an effect that may be mediated by FXR (225).

A study has shown that licorice extract improves IR, endotoxemia-related colonic inflammation and serum lipids in diabetic mice. In addition, licorice extract reshapes the intestinal flora by reducing *Lachnospiraceae_NK4A136_group*, while increasing *Akkermansia* and *Bacteroides*.These results indicate that the modulation of intestinal microbiota and colonic TLR4/NF-κB signaling pathway in diabetic mice may be the main reason for the anti-diabetic effect of licorice extracts (226). Studies have shown that the antidiabetic effect of Scutellaria-coptis herb couple (SC), one of the famous herbal compounds in traditional herbal combinations for diabetes treatment, is attributed to its modulation of gut microbiota and anti-inflammatory effects involving TLR4 signaling pathway (227). A rich-polyphenols extract of *Dendrobium loddigesii* (DJP) has been used to treat diabetic db/db mice, possibly due to the effects of DJP-induced reduction of inflammation and oxidative stress and improved intestinal flora balance, resulting in improved diabetic symptoms and complications in mice (228). Gegen Qinlian Decoction (GQD), a traditional Chinese medicine formula, has already been applied to treat common metabolic diseases such as T2DM. The mechanism of GQD for T2DM is mainly through altering the structure of the entire intestinal microbiota, enriching it with many butyrate-producing bacteria, thereby reducing intestinal inflammation and lowering blood glucose (229). Berberine, a hypothetical key active pharmaceutical ingredient of GQD, is also isolated from rhizoma coptidis and acts as an active alkaloid to achieve pharmacological effects by regulating the intestinal microbiota (230). In animal models of T2DM, berberine has been shown to increase the number of beneficial bacteria, reduce potentially pathogenic bacteria, and protect the islets and protect insulin target organs by reducing the invasion of inflammatory cells and inhibiting the development of a systemic inflammatory response (231). In a mouse model with T2DM, ginsenosides have been shown to modulate the intestinal flora, thereby reducing gut mucosal damage and a range of inflammatory responses (232, 233). Purified citrus polymethoxyflavone-rich extract (PMFE) significantly increases the abundance of *Bacteroides ovatus*, *Bacteroides uniformis* and *Bacteroides thetaiotaomicron*. The enrichment of *Bacteroides ovatus* by PMFE contributes to lower BCAA levels, weight loss and MetS relief (234). Ginsenoside Rb1 (Rb1) significantly changes the composition of the intestinal microbiota as it significantly increases the abundance of the bacterium *Akkermansia* spp. to which circulating alanine levels are related. Modulation of alanine may be correlated with *Akkermansia* spp., which is elevated in abundance by Rb1 to gain glucose homeostasis (235).

The role of herbal medicines in improving the intestinal microbiota in the treatment of T2DM deserves recognition. However, from the perspective of modern science, the underlying mechanism requires more explorations. Most of the effects of herbal medicines on microbiota are associated with intestinal flora structure modulation, increased richness of beneficial bacteria and butyrate concentration in gut, inhibition of opportunistic pathogens. Most of the findings are based on animal experiments, thus indicating the need of further evidence from human studies, and the underlying principles remain to be explored.

### 5.4 Diet and exercise

Studies have shown that weight loss of approximately 15 kg achieved through calorie restriction resulted in T2DM remission in 80% of obese and T2DM individuals. In addition, this remission is proportional to the amount of weight loss (236). Therefore, optimizing carbohydrate intake and increasing dietary fiber supplementation is particularly important for patients with T2DM. Fiber has already been recognized to play a critical role in regulating metabolism and preventing chronic gastroenterological diseases (237). The lower fiber content in the Western diet (especially in industrialized countries) is closely related to the elevated prevalence of metabolic disease states. Thus, alteration of the microbiota through dietary fiber interventions can improve health. Associations have been found between plant-based diets and taxa, such as *Roseburia*, *Faecalibacterium prausnitzii* and *Eubacterium rectale*, as well as an elevation in total SCFAs (238). SCFAs are produced by microbial fermentation of dietary fiber, which have cholesterol-lowering and glucose-control effects (239). Dietary fiber has been identified to markedly increase the RA of *Bifidobacterium* and total SCFAs while reducing glycated hemoglobin (240). Another study has reported that *Bifidobacterium pseudocatenulatum*, an acetate producer, is one of the most significantly promoted SCFA-produced microbiota by dietary fibers, and inoculation with this strain results in improved IR and postprandial glycemic response (31). A previous study has also shown that an almond-based low carbohydrate diet may improve glucose metabolism in patients with T2DM by increasing SCFA-producing bacteria, including *Roseburia*, *Ruminococcus* and *Eubacterium*, to elevate SCFA production and activate GPR43 to sustain GLP-1 secretion (48). Consistently, another study has suggested that almond-based diets may promote SCFA-producing bacteria while decreasing hemoglobin and body mass index (BMI) in T2DM individuals (241). The Green-Mediterranean diet with a gradual increase in plant components induces specific alterations in the intestinal flora and BCAA metabolism, including an elevation in *Prevotella* abundance and BCAA degradation as well as a decrease in *Bifidobacterium* abundance and BCAA biosynthesis, leading to increased insulin sensitivity (242).

Similarly, increasing physical activity and fitness is another important factor in alleviating T2DM. Physical activity is essential for reducing blood glucose and improving insulin sensitivity (243). A previous study has shown that exercise increases the abundance of *Akkermansia muciniphila* in the intestinal flora of athletes and increases the diversity of the intestinal microbiota (244). Regular exercise has also been reported to influence the composition of intestinal flora, elevate the production of SCFAs and plasma SCFA concentrations to ameliorate IR in skeletal muscle (245). Furthermore, exercise-induced elevation of SCFA-producing bacteria and improvement of intestinal barrier integrity play critical roles in T2DM improvement (246). Studies have shown that taxa producing SCFAs are positively correlated with changes in locomotion and mass, indicating that SCFAs may be involved in improving locomotor performance (247, 248).

## 6 Conclusion and future perspective

The recognized risk factors for the development of diabetes include the interaction among different elements such as genetic susceptibility, diet, physical activity, smoking, and stress. The interplay of diet and gut microbiota determines the formation and absorption of different metabolites. It has been found that T2DM individuals can be divided into different clusters depending on characteristics of patients and risk of developing complications. Differences in pathophysiology of various groups in the pathogenesis were also found in individuals with prediabetes (249). Different patient subgroups may respond to the same treatment in very different ways. Thus, early identification of patients with diverse traits of T2DM has allowed us to predict the disease progression and response of individuals to the corresponding treatment regimen (250).

Only recently has the importance of the gut microbiota been more widely recognized. Bacteria that live in symbiosis with humans play a critical part in health and disease, and this makes microbiology one of the most active frontiers in biomedicine today. The microbial “organ” of the intestinal flora has involved in the regulation of human health and metabolism (251).Numerous animal studies and clinical trials have strongly supported the role of intestinal flora in obesity, IR and T2DM, as well as the proposition that the gut microbiome may influence a range of host systems and metabolic pathways through the production of metabolites (39, 40, 77, 252, 253). As mentioned before, *Bacteroidetes*, *Firmicutes*, *Ruminococcus torques*, and other species regulate changes in BAs. *Akkermansia muciniphila*, *Roseburia* spp., *Prevotella* spp., and others play important roles in the production of SCAAs. *Prevotella copri* and *Bacteroides vulgatus* are closely related to the biosynthesis of BCAAs. The metabolites of these microorganisms are highly associated with the evolution of obesity and T2DM in humans. However, changes in other microbial metabolites, such as imidazole propionate, indole, and TMAO, also play an important role in T2DM, but the corresponding clinical evidences are not sufficient.

Microbially directed interventions for diabetes can be divided into nontargeted and targeted therapies. Nontargeted interventions include exercise, personalized nutrition, Probiotics and FMT, which act on the overall improvement of flora composition and function. Targeted interventions include engineered microorganisms and drugs that target the metabolism of specific microorganisms, which act on specific changes in the metabolism-related flora. Numerous animal experiments and clinical trials have shown that groups with distinct base states respond very differently to therapeutic measures targeting the gut microbiota (45, 46). This discrepancy may be closely related to the composition of the microbial flora present in the organism itself. Therefore, an important prerequisite for the application of microbial therapies is the clarification of the pathogenic mechanism and the baseline microbial level of the organism. It is also the basis for successfully colonizing the transplanted microorganisms in an individualized manner. Overall, by analyzing the causal relationship between T2DM and gut flora, we conclude that gut flora can be used not only as a diagnostic biomarker but also as a promising therapeutic target for T2DM. The utilization of gut microbiota may contribute to a more precise and personalized treatment of T2DM patients.

## Author contributions

Conceptualization: YW, GR. Investigation: LL, JZ, GR, YC, MZ, and ZX. Writing—original draft preparation: LL, JZ, and YW. Writing—review and editing: LL, JZ, GR, YC, and MZ. Supervision: YW, GR. All authors contributed to the article and approved the submitted version.

## Funding

This work was funded by grants from the National Natural Science Foundation of China (NSFC 82070571), Chongqing Science and Health Joint Project (2022MSXM052), and Army Medical Center Military Medical Frontier Innovation Capability Program (CX2019JS222).

## Acknowledgments

We thank Servier Medical Art (http://smart.servier.com/) for the reference of Figures in this review, licensed under a Creative Common Attribution 3.0 Generic License (https://creativecommons.org/licenses/by/3.0/).

## Conflict of interest

The authors declare that the research was conducted in the absence of any commercial or financial relationships that could be construed as a potential conflict of interest.

## Publisher’s note

All claims expressed in this article are solely those of the authors and do not necessarily represent those of their affiliated organizations, or those of the publisher, the editors and the reviewers. Any product that may be evaluated in this article, or claim that may be made by its manufacturer, is not guaranteed or endorsed by the publisher.
